# Use of Damage Control Surgery and Resuscitative Endovascular Balloon Occlusion of the Aorta (REBOA) in Penetrating Torso Trauma: A Systematic Review

**DOI:** 10.7759/cureus.92082

**Published:** 2025-09-11

**Authors:** Mohamed K Abouelsadat, Mahetab Shehata, Abdelrahman Ibrahim, Ahmed Soliman, Mohammad Hussien, Shenouda Shehata Abdelmesih, Mohammed Jibreel Mohammed, Anwar Al-Kassar, Sharmila Venkatachalapathi, Mawada Taha, Nadia Malik

**Affiliations:** 1 Vascular Surgery, Royal Free Hospital, London, GBR; 2 Vascular Surgery, Norfolk and Norwich University Hospital, Norwich, GBR; 3 Trauma and Orthopaedics, University Hospitals of North Midlands NHS Trust, Stoke-on-Trent, GBR; 4 Vascular Surgery, York and Scarborough Teaching Hospitals NHS Foundation Trust, Scarborough, GBR; 5 Orthopaedics and Traumatology, Khoula Hospital, Muscat, OMN; 6 Vascular Surgery, Countess of Chester Hospital, Chester, GBR; 7 Internal Medicine, Government Periyar Hospital, Mayiladuthurai, Mayiladuthurai, IND; 8 Internal Medicine, Vadamalayan Hospitals Pvt Ltd., Dindigul, IND; 9 General Surgery, National Ribat University, Khartoum, SDN; 10 Medicine, National Medical Center, Lahore, PAK

**Keywords:** damage control surgery, hemorrhagic shock, penetrating torso trauma, resuscitative endovascular balloon occlusion of the aorta (reboa), systematic review

## Abstract

Penetrating torso trauma, often complicated by non-compressible torso hemorrhage (NCTH), is a leading cause of preventable death. Damage control surgery (DCS) is standard care, with resuscitative endovascular balloon occlusion of the aorta (REBOA) emerging as a minimally invasive adjunct for temporary hemorrhage control. This Preferred Reporting Items for Systematic Reviews and Meta-Analyses (PRISMA)-guided systematic review included six studies with a total of 2,705 patients, comparing combined DCS and REBOA with DCS alone or alternative strategies. Several studies indicated improved survival and faster hemorrhage control, while others reported higher control rates compared to resuscitative thoracotomy. Conversely, some data suggested increased mortality and complications in penetrating abdominal vascular injuries. Pooled findings suggested a potential survival benefit, but heterogeneity and confounding limited certainty. Reported risks included ischemia-reperfusion injury, limb ischemia, and increased transfusion requirements. REBOA may improve outcomes in selected patients, but its benefit appears context-dependent, warranting prospective trials to refine indications and timing.

## Introduction and background

The torso, also known as the trunk, refers to the central or main part of the body in humans, including the chest, abdomen, pelvis, and upper and lower back. On average, road accidents cause 3,406 deaths worldwide [1]. Road traffic accidents (RTAs) represent the leading cause of torso injuries, specifically in individuals under the age of 45. Penetrating torso trauma represents a severe and often fatal category of traumatic injuries, contributing substantially to both pre-hospital and in-hospital mortality across the globe. This type of trauma is frequently associated with catastrophic internal bleeding that cannot be controlled through external compression. Non-compressible torso hemorrhage alone is estimated to be responsible for nearly 90% of all exsanguination-related deaths in trauma patients [2]. Damage control surgery (DCS) has emerged as a pivotal strategy in the management of such critically injured patients. Its guiding principles involve abbreviated surgical intervention focused on immediate control of bleeding and contamination, followed by intensive care unit (ICU) resuscitation and delayed definitive repair once physiological stability is restored. While DCS has improved survival, outcomes in patients with profound hemorrhagic shock remain suboptimal, prompting interest in adjunctive technologies to bridge the gap to definitive hemostasis.

However, resuscitative endovascular balloon occlusion of the aorta (REBOA) is an advanced, minimally invasive endovascular technique designed to temporarily control life-threatening hemorrhage below the level of the diaphragm while maintaining perfusion to critical organs. Specific clinical indications include severe abdominal or pelvic bleeding (e.g., from pelvic fractures or vascular injuries of iliac or femoral origin) and junctional hemorrhage not amenable to external compression. However, before initiating REBOA, clinicians must rapidly rule out bleeding proximal to the intended occlusion zone, such as intrathoracic hemorrhage, pericardial tamponade, or mediastinal injury, often via chest imaging or focused assessment with sonography in trauma (FAST) ultrasound, since REBOA in such contexts might worsen bleeding or delay critical thoracotomy [3]. REBOA has evolved into a valuable adjunct in modern trauma and acute care settings, particularly for patients with non-compressible torso hemorrhage who are at imminent risk of exsanguination [4].

The procedure involves percutaneous or surgically assisted insertion of a specialized balloon-tipped endovascular catheter through the common femoral artery (CFA). Under fluoroscopic, ultrasound, or landmark-guided techniques, the catheter is advanced into the aorta and positioned in a specific zone based on the source and location of bleeding. Zone I, located in the descending thoracic aorta between the left subclavian artery and the celiac artery, is typically selected for severe intra-abdominal or retroperitoneal hemorrhage. Zone III, situated in the infra-renal abdominal aorta between the lowest renal artery and the aortic bifurcation, is preferred for life-threatening pelvic or junctional bleeding. (Zone II, which is between the celiac and lowest renal arteries, is generally avoided due to the risk of compromising mesenteric perfusion without effective hemorrhage control.) Once positioned, the balloon is inflated to achieve complete or partial occlusion of the aortic lumen. This mechanical occlusion halts distal hemorrhage, significantly increases proximal aortic pressure, and enhances perfusion to the heart and brain, thereby buying critical time for definitive surgical or endovascular repair. The duration of occlusion is carefully limited, typically to under 30-60 minutes in Zone I to reduce the risk of ischemic injury to distal organs and tissues [5].

Despite its promise, REBOA carries limitations and risks, including technical difficulty in obtaining arterial access in hypovolemic patients, ischemia-reperfusion injury, limb ischemia, acute kidney injury, vascular injury, and the potential for delayed definitive hemorrhage control. Its efficacy is further influenced by operator experience, institutional readiness, and strict adherence to time limits for occlusion to minimize ischemic complications. Given the high mortality associated with penetrating torso trauma and the evolving but limited evidence base for REBOA as an adjunct to DCS, there is a need for a structured synthesis of current literature. This systematic review aims to critically evaluate and synthesize available evidence on the combined use of DCS and REBOA in penetrating torso trauma. The focus is on assessing their combined impact on hemodynamic stabilization, survival outcomes, and complication profiles, while identifying knowledge gaps and informing future clinical protocols and research priorities.

## Review

Materials and methods

Search Strategy

Following the Preferred Reporting Items for Systematic Reviews and Meta-Analyses (PRISMA) 2020 framework, a comprehensive and systematic search strategy was employed to maximize the retrieval of relevant literature [6]. Four major biomedical databases, such as PubMed/MEDLINE, Embase, Scopus, and the Cochrane Library, were queried from their inception through July 2025. The search incorporated both controlled vocabulary terms (Medical Subject Headings (MeSH) in PubMed and Emtree terms in Embase) and free-text keywords to ensure broad coverage of the topic. The primary search terms included “damage control surgery,” “resuscitative endovascular balloon occlusion of the aorta,” “REBOA,” “penetrating torso trauma,” “hemorrhagic shock,” and related synonyms. Boolean operators (“AND,” “OR”) and truncation symbols were utilized to refine the search for an optimal balance between sensitivity and specificity. Filters were applied to restrict results to human studies, English-language publications, and peer-reviewed articles. The strategy was iteratively refined after preliminary searches to capture emerging terminology and to ensure inclusion of all potentially eligible studies relevant to the combined application of DCS and REBOA in penetrating torso trauma.

Eligibility Criteria

The inclusion and exclusion criteria for this systematic review were established using the Population, Intervention, Comparator, Outcomes (PICO) framework to ensure methodological clarity and relevance [7]. The population (P) of interest comprised human subjects presenting with penetrating torso trauma complicated by hemorrhagic shock. The intervention (I) was the combined application of DCS and REBOA. The comparator (C) included patients managed with DCS alone, either with or without alternative resuscitative measures, or those undergoing other hemorrhage control strategies. The outcomes (O) of interest were hemodynamic stabilization, survival rates, and procedure-related complications, including ischemic events. Eligible studies met all of the following criteria: (1) human studies evaluating DCS with REBOA in penetrating torso trauma, (2) comparative or descriptive designs with outcome data, (3) full-text articles published in English, (4) publication prior to June 2025, and (5) reporting defined hemodynamic or survival endpoints. Studies were excluded if they (1) were single-patient case reports, (2) involved animal or cadaveric models, (3) were editorials or conference abstracts lacking complete datasets, or (4) failed to report explicit outcome measures.

Data Extraction

Data were systematically collected using predesigned extraction templates, documenting study authors, publication year, sample size, intervention details, comparators, measured outcomes, and procedural specifics. Two reviewers independently performed the extraction to ensure accuracy and completeness. All extracted information underwent cross-verification, and any discrepancies were addressed through discussion until agreement was reached. This process ensured consistency and minimized the risk of data misinterpretation.

Risk of Bias Assessment

Observational studies and retrospective cohort studies were appraised with the Newcastle-Ottawa Scale [8], and meta-analyses were appraised with the Risk of Bias in Systematic Reviews (ROBIS) scale [9]. Each assessment was conducted independently by two reviewers to maintain objectivity. A cross-checking process was implemented to confirm scoring accuracy. Disagreements were resolved through consensus to ensure a reliable risk of bias evaluation.

Data Synthesis

Given the substantial clinical and methodological heterogeneity among studies, a narrative synthesis was undertaken. Meta-analysis was not feasible due to variability in study designs, populations, and outcome measures. Key trends in hemodynamic stabilization, survival rates, and complication profiles were systematically summarized. Findings were interpreted qualitatively to highlight patterns and clinical implications.

Results

Study Selection Process

**Figure 1:**
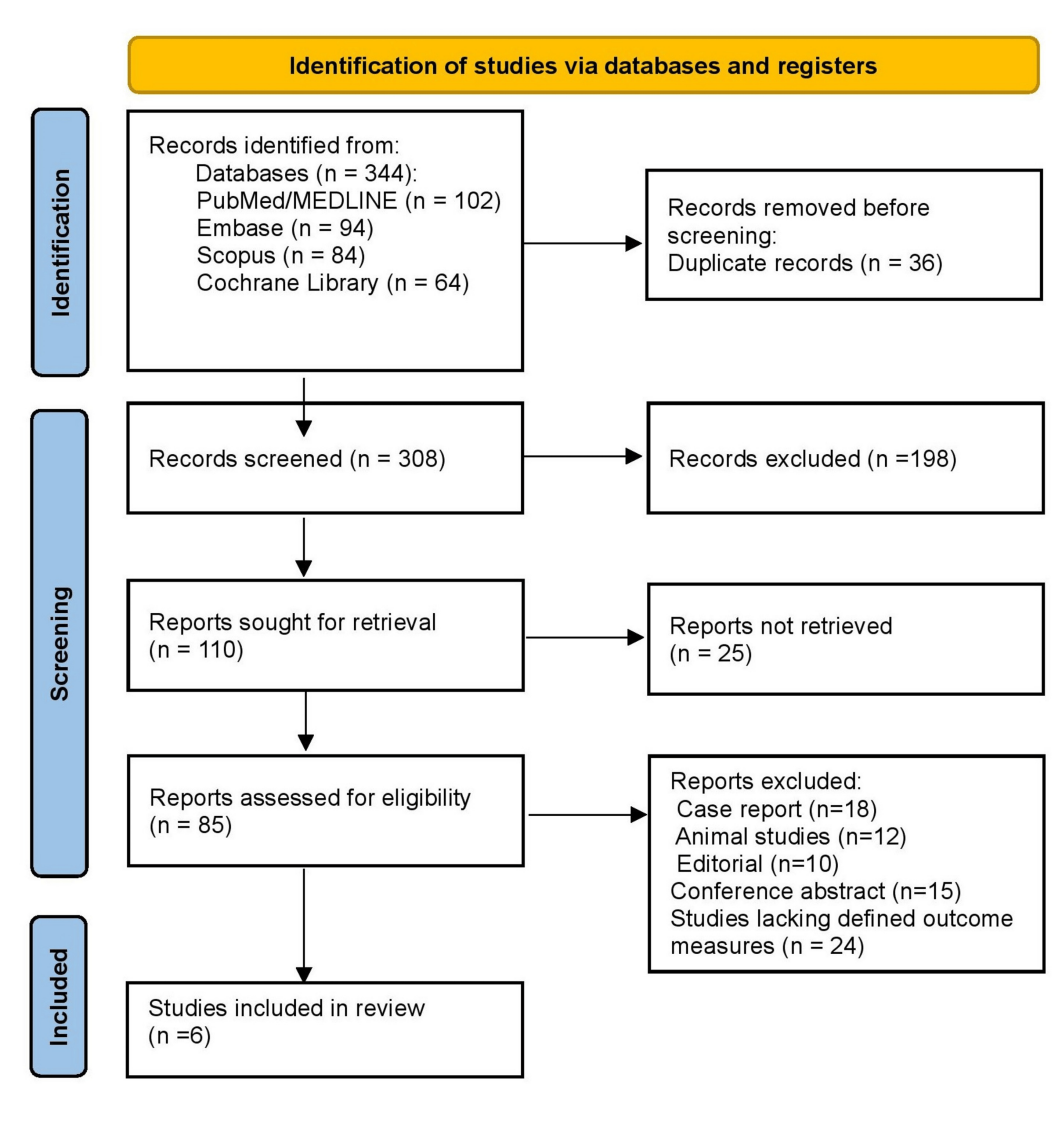
The PRISMA 2020 flow diagram detailing the study selection process PRISMA: Preferred Reporting Items for Systematic Reviews and Meta-Analyses

Figure 1 represents the PRISMA 2020 flow diagram detailing the study selection process. A total of 344 records were identified from database searches, including PubMed/MEDLINE (n = 102), Embase (n = 94), Scopus (n = 84), and the Cochrane Library (n = 64). Following the removal of 36 duplicate records, 308 records underwent title and abstract screening, of which 198 were excluded for irrelevance. Reports sought for retrieval numbered 110, and all were successfully retrieved. Subsequent full-text assessment of 85 reports led to the exclusion of 79 studies: case reports (n = 18), animal studies (n = 12), editorials (n = 10), conference abstracts without full data (n = 15), and studies lacking defined outcome measures (n = 24). Ultimately, six studies fulfilled the eligibility criteria and were included in the final review.

Characteristics of the Selected Studies

As detailed in Table 1, the six studies involving 2,705 patients with penetrating or severe torso trauma, REBOA showed variable outcomes. Some studies reported improved survival and faster hemorrhage control compared to DCS or resuscitative thoracotomy (RT), while others found increased complications and mortality. Overall, REBOA can be effective in selected patients, but its benefits are highly context-dependent.

**Table 1:** Characteristics of the selected studies REBOA: resuscitative endovascular balloon occlusion of the aorta; DCS: damage control surgery; RT: resuscitative thoracotomy; ISS: Injury Severity Score (is a numerical score that quantifies the overall severity of a trauma patient’s injuries to predict mortality and outcomes); aOR: adjusted odds ratio (is the measure of association between an exposure and outcome, accounting for other influencing factors); BP: blood pressure; NCTH: non-compressible torso hemorrhage

Authors and Year	Population (P)	Exposure (I)	Comparator (C)	Outcomes (O)	Pathophysiological Findings	Anatomical Impact	Hemorrhagic Control
García et al. (2021) [10]	345 penetrating torso trauma patients (28 received REBOA)	DCS + REBOA	DCS alone (no REBOA)	Mortality, hemodynamic outcomes	Adjusted mortality is lower with REBOA	Torso hemorrhage	Effective in reducing hemorrhage
Otsuka et al. (2018) [11]	107severe torso trauma patients (ISS>16; 15 REBOA, 92 no REBOA)	REBOA + resuscitative hemostasis	No REBOA	In-hospital mortality, time to hemostasis	REBOA associated with survival benefit (aOR 7.43)	Multi-torso hemorrhage	Hemorrhage controlled faster
Balch et al. (2023) [12]	26 REBOA vs. 13 RT patients with blunt or penetrating torso trauma	REBOA (Zone I)	Resuscitative thoracotomy	Survival to discharge, BP	REBOA achieved hemorrhage control in 77% vs. 8% in RT	Blunt and penetrating	Better proximal hemostasis
Koh et al. (2023) [13]	72 traumatic cardiac arrest patients (26 REBOA, 46 RT)	REBOA	Resuscitative thoracotomy	Mortality, transfusion needs	Similar survival, longer time to occlusion, and more transfusions with REBOA	NCTH in arrest	Controlled hemorrhage, but slower
Manzano et al. (2017) [14]	1276 pooled observational data	REBOA	RT or no REBOA	Mortality outcomes	REBOA improved survival across NCTH	Broad torso injuries	Generally effective temporizing
Nekooei et al. (2024) [15]	879 penetrating abdominal vascular injury patients	REBOA	No REBOA	In-hospital mortality, complication rates	REBOA is associated with increased mortality and complications	Abdominal vascular sites	Temporizing, but higher risk

Risk of Bias Assessment

As detailed in Table 2, the risk of bias varied across the six studies. García et al. (2021) [10] had a moderate risk due to a small REBOA subgroup and possible confounding by indication. Otsuka et al. (2018) [11] showed moderate-high risk from a small sample size, single-center design, and likely selection bias. Balch et al. (2023) [12] had a moderate risk, benefiting from registry data but limited by heterogeneity and comparator differences. Koh et al. (2023) [13] were at high risk, with small, highly selected cardiac arrest cases and potential measurement bias. Manzano et al. (2017) [14] had a moderate risk from pooling heterogeneous observational data, and Nekooei et al. (2024) [15] had a moderate-high risk due to registry coding limitations and residual confounding despite adjustment.

**Table 2:** Risk of bias assessment REBOA: resuscitative endovascular balloon occlusion of the aorta; RT: resuscitative thoracotomy; NOS: Newcastle-Ottawa Scale; confounding by indication: bias occurring when sicker patients are more likely to receive a particular treatment, affecting outcomes; sample size: number of patients included in a study or subgroup; registry data: data collected systematically from patient registries or hospital databases

Study	Study Design	Risk of Bias Tool	Risk of Bias Rating	Justification
García et al. (2021) [10]	Retrospective cohort	NOS	Moderate	Reasonable selection of a trauma cohort, but small REBOA subgroup (n=28) increases imprecision; potential for confounding by indication (sicker patients more likely to receive REBOA) despite adjustment; outcome ascertainment from registry/hospital data likely adequate.
Otsuka et al. (2018) [11]	Retrospective single-center cohort	NOS	Moderate-High	Small overall sample and very small REBOA arm (n=15) limit external validity and precision; selection and performance biases likely (non-random allocation); some adjustment reported, but residual confounding plausible.
Balch et al. (2023) [12]	Registry/comparative cohort (REBOA vs. RT)	NOS	Moderate	Use of registry data improves sample ascertainment but suffers from heterogeneity of indications and center practices; comparator (RT) may represent a different severity spectrum; outcome definitions appear clear, but temporality and follow-up completeness are variable.
Koh et al. (2023) [13]	Retrospective cohort (traumatic cardiac arrest subgroup)	NOS	High	Highly selected, critically ill population with major confounding by indication and timing; small sample sizes increase chance findings; measurement bias for time-to-occlusion and transfusion volumes is likely; limited adjustment for important prognostic covariates.
Manzano et al. (2017) [14]	Pooled observational data/meta-analysis of cohorts	Adapted NOS/ROBIS considerations	Moderate	Aggregates multiple observational datasets - improves overall power but inherits heterogeneity and publication bias from components; variable methodological quality across included cohorts reduces certainty; sensitivity analyses are partially reported.
Nekooei et al. (2024) [15]	Large retrospective cohort/database analysis	NOS	Moderate-High	Large sample size increases precision, but reliance on administrative/registry coding can introduce misclassification; despite propensity adjustment, residual confounding (severity, physiologic variables) remains possible; outcomes and complication reporting are likely robust but not immune to under-ascertainment.

Discussion

Torso involves the chest, abdomen, and pelvis. The most common cause of injury to the torso is trauma/RTA. Approximately 39% of trauma accounts for torso trauma. Torso trauma has an incidence rate of 488/100,000 person/year. It is more commonly involved in people under the age of 45 and comparatively, more in males than females. Chest trauma, which is presented to the emergency department, accounts for 10% of admissions and 50% of deaths. In case of abdominal trauma, only 25% need surgical exploration and damage control [16]. Patients with penetrating torso trauma often arrive in a profoundly unstable state, as uncontrolled non-compressible hemorrhage can rapidly precipitate massive blood loss, coagulopathy, metabolic acidosis, and hypothermia - the classic “lethal triad” that accelerates early mortality. In such scenarios, rapid recognition and intervention are critical, with the ABCDE primary survey serving as the structured framework for early trauma care. Airway (A) is assessed first to ensure patency, with prompt intubation or surgical airway if compromise is likely. Breathing (B) follows, focusing on identifying and correcting life-threatening thoracic injuries such as tension pneumothorax or massive hemothorax through decompression, chest tube insertion, or ventilation support. Circulation (C) prioritizes immediate hemorrhage control and shock reversal through direct pressure, rapid transfusion, or advanced interventions such as REBOA. Disability (D) entails a rapid neurological evaluation, often via Glasgow Coma Scale scoring, to detect and manage brain or spinal injury while addressing contributing factors like hypoxia and hypoperfusion. Finally, exposure (E) requires full visualization of the patient to locate all injuries while implementing active warming strategies to counter hypothermia. This systematic, prioritized approach ensures that the most immediate threats to life are addressed in order, improving the likelihood of survival and enabling a timely transition to definitive surgical care [17].

In cases of severe torso trauma complicated by hemorrhagic shock, the cornerstone of management lies in the rapid achievement of bleeding control coupled with targeted physiologic resuscitation. Early priorities include prompt identification of the hemorrhage source and institution of temporizing or definitive interventions to halt ongoing blood loss. Resuscitative strategies are increasingly guided by damage control principles, incorporating permissive hypotension to minimize clot disruption in the absence of traumatic brain injury, alongside balanced transfusion protocols delivering red blood cells, plasma, and platelets in ratios approximating whole blood. Correction of coagulopathy, whether dilutional, consumptive, or trauma-induced, is addressed through early administration of fibrinogen, tranexamic acid, and judicious temperature management to mitigate the effects of hypothermia on clot function [18].

While definitive surgical repair is the gold standard for achieving complete hemorrhage control in severe torso trauma, profoundly unstable patients often cannot withstand the metabolic, inflammatory, and hemodynamic burden of prolonged operative interventions. In such cases, DCS is employed as a staged, physiology-preserving strategy. The initial operation, whether laparotomy, thoracotomy, or hybrid endovascular-open approach, is deliberately abbreviated to focus solely on rapid hemorrhage arrest (e.g., vascular clamping, ligation, packing, temporary shunting) and contamination control (e.g., stapled bowel resection without anastomosis). This phase is typically limited to 60-90 minutes to reduce operative stress and prevent worsening shock [19].

Following this initial phase, the patient is transferred to the ICU for damage control resuscitation (DCR), targeting correction of hypothermia, reversal of acidosis, and restoration of coagulation competence. This includes balanced transfusion strategies (often 1:1:1 red blood cells, plasma, and platelets), fibrinogen replacement, antifibrinolytic therapy such as tranexamic acid, and active warming measures. Hemodynamic optimization is prioritized to restore tissue perfusion and support organ function. Once physiologic stability is achieved, indicated by normalized pH, lactate clearance, stable core temperature, and effective hemostasis, the patient undergoes definitive anatomical repair in a planned return to the operating room [20]. This approach interrupts the lethal triad of hypothermia, acidosis, and coagulopathy, improving survival in carefully selected patients, particularly when combined with modern adjuncts such as REBOA and hybrid trauma operating suites that enable seamless surgical and endovascular transitions. REBOA is an endovascular adjunct to temporize subdiaphragmatic hemorrhage and improve central perfusion. Indications include exsanguinating pelvic or intra-abdominal hemorrhage unresponsive to resuscitation, junctional bleeding not amenable to compression, and selected traumatic cardiac arrests. The technique involves femoral artery cannulation, advancement to Zone I or Zone III, and controlled balloon inflation, ideally limited to <60 minutes in Zone I to reduce ischemia. Limitations of REBOA include difficulty achieving access in severe shock, vascular complications, distal ischemia, reperfusion injury, potential worsening of proximal bleeding, and the need for rapid definitive hemorrhage control. Prolonged occlusion increases multi-organ injury risk, making strict selection and operator training critical [21].

The current body of evidence regarding the use of REBOA in penetrating torso trauma demonstrates a complex and sometimes contradictory picture. In a multicenter retrospective analysis, García et al. (2021) [10] observed a reduction in adjusted mortality when REBOA was employed alongside DCS, suggesting that rapid aortic occlusion may enhance central perfusion during the critical pre-definitive repair phase. Similarly, Otsuka et al. (2018) [11], in a smaller single-center study, reported a survival benefit and faster achievement of hemorrhage control in patients undergoing REBOA-assisted resuscitative hemostasis, indicating potential utility in expediting stabilization. However, other findings temper this optimism. Balch et al. (2023) [12] demonstrated that REBOA achieved markedly higher rates of proximal hemorrhage control compared to RT, but Koh et al. (2023) [13] identified operational drawbacks, including prolonged time to aortic occlusion and increased transfusion requirements in traumatic cardiac arrest patients, raising concerns about delays in critical perfusion restoration.

Broader analyses also reflect this variability: a pooled observational synthesis by Manzano et al. (2017) [14] suggested an overall survival benefit across non-compressible torso hemorrhage cohorts, yet a large database study by Nekooei et al. (2024) [15] associated REBOA use with higher mortality and complication rates, particularly in penetrating abdominal vascular injuries. The variability in reported outcomes highlights that the effectiveness of REBOA is highly dependent on clinical context. Its hemodynamic advantages are most likely to be realized in carefully selected, physiologically salvageable patients who can be transitioned rapidly to definitive hemorrhage control. In contrast, indiscriminate application or delays in surgical intervention may diminish or even reverse its potential benefits. Alternative strategies, such as RT with aortic cross-clamping, pre-peritoneal pelvic packing, hybrid open endovascular procedures, and early angioembolization, remain important options, with the optimal choice guided by the patient’s injury pattern, physiological status, and the institution’s resources and expertise.

At present, the body of evidence for REBOA and DCS in penetrating torso trauma is largely observational, often constrained by small cohorts, heterogeneity in injury mechanisms, and significant variation in institutional protocols. Common methodological limitations include confounding by indication, inconsistent outcome definitions, and incomplete documentation of complication rates, all of which hinder robust comparative assessment. Moreover, few studies evaluate long-term survival or functional outcomes, further restricting the ability to establish durable benefit. These limitations reduce generalizability and underscore the pressing need for well-designed, prospective, multi-center clinical trials aimed at refining patient selection, optimizing procedural timing, and standardizing perioperative management to maximize both survival and quality of recovery.

## Conclusions

REBOA is a valuable, minimally invasive tool for controlling non-compressible torso hemorrhage by temporarily occluding the aorta and preserving blood flow to vital organs. It acts as a critical bridge to definitive surgical management, often within the framework of DCS, which focuses on rapid hemorrhage control and staged repair to improve survival in severe trauma. Successful use of REBOA requires careful patient selection and multidisciplinary expertise to avoid complications. Trauma centers should adopt standardized training and protocols integrating REBOA into DCR. Furthermore, close monitoring during and after balloon occlusion is essential to minimize ischemic injury and reperfusion-related complications. As technology advances, refinement of partial occlusion techniques may further enhance their safety and effectiveness. Ongoing research is needed to optimize its application and improve outcomes.
